# Relationship between differentially expressed mRNA and mRNA-protein correlations in a xenograft model system

**DOI:** 10.1038/srep10775

**Published:** 2015-06-08

**Authors:** Antonis Koussounadis, Simon P. Langdon, In Hwa Um, David J. Harrison, V. Anne Smith

**Affiliations:** 1 School of Biology, University of St Andrews, St Andrews, Fife, KY16 9TH, UK; 2 Division of Pathology, University of Edinburgh, Edinburgh, EH4 2XU, UK; 3 School of Medicine, University of St Andrews, St Andrews, Fife, KY16 9TF, UK

## Abstract

Differential mRNA expression studies implicitly assume that changes in mRNA expression have biological meaning, most likely mediated by corresponding changes in protein levels. Yet studies into mRNA-protein correspondence have shown notoriously poor correlation between mRNA and protein expression levels, creating concern for inferences from only mRNA expression data. However, none of these studies have examined in particular differentially expressed mRNA. Here, we examined this question in an ovarian cancer xenograft model. We measured protein and mRNA expression for twenty-nine genes in four drug-treatment conditions and in untreated controls. We identified mRNAs differentially expressed between drug-treated xenografts and controls, then analysed mRNA-protein expression correlation across a five-point time-course within each of the four experimental conditions. We evaluated correlations between mRNAs and their protein products for mRNAs differentially expressed within an experimental condition compared to those that are not. We found that differentially expressed mRNAs correlate significantly better with their protein product than non-differentially expressed mRNAs. This result increases confidence for the use of differential mRNA expression for biological discovery in this system, as well as providing optimism for the usefulness of inferences from mRNA expression in general.

Genome-wide correlation between expression levels of mRNA and protein are notoriously poor, hovering around 40% explanatory power across many studies^1^,^2^. The discrepancy is typically attributed to other levels of regulation between transcript and protein product^3^. However, mRNA expression and, in particular, significant differences in mRNA expression between conditions, is commonly used for biological discovery. For example, mRNA assays are one of the more promising techniques for novel diagnostics of cancer^4^. This reliance on mRNA measurements can be traced both to the relative ease of availability of mRNA compared to protein data^2^,^3^, as well as to the apparently sensible assumption that differential expression ought to *mean something*, i.e., translate into a functional difference between conditions—and this functional difference is typically attributed to protein action. Thus, despite the known poor overall correlation between mRNAs and their protein products, there is an implicit assumption that differentially expressed mRNAs impact their respective experimental conditions via differences in proteins. However, to the best of our knowledge, this assumption has never been explicitly tested.

Most studies of mRNA-protein expression correspondence examine the question from a genome-wide viewpoint. These genome-wide studies calculate a correlation coefficient (or other correspondence metric) of mRNA expression versus the corresponding protein expression across a large number of genes (e.g.,^5^,^6^,^7^,^8^,^9^,^10^,^11^,^12^,^13^,^14^,^15^,^16^,^17^,^18^,^19^,^20^; Fig. 1a). Some studies have noted that certain classes of genes have higher correlations, often those for which reasons to expect tight synchrony can be generated: e.g., secreted proteins, which would only require transcription when needed (human plasma proteins^21^, yeast pheromones^12^); cell cycle genes, which are time-dependent (in humans^20^, in yeast^9^); and bacterial genes optimised for fast translation (optimal codon-containing genes^13^, genes within short operons^18^). Only a limited number of studies have investigated correspondence for individual genes across samples or experimental conditions^6^,^11^,^13^,^15^,^18^,^22^,^23^,^24^,^25^,^26^,^27^,^28^,^29^ (Fig. 1b). Of these individual gene studies, some simply report the range of correlation coefficients to make the point that mRNA and protein expression are sometimes discordant^22^,^26^,^28^,^29^; some cluster or perform similar analyses on expression profiles across samples, finding groups of discordant and concordant mRNA-protein pairs^11^,^13^,^15^,^18^,^24^,^30^; and others identify differentially expressed proteins and examine the corresponding mRNA expression, typically finding 0-50% of the mRNA levels concordant^6^,^23^,^24^,^25^,^27^. This last analysis asks whether differentially expressed proteins are the result of mRNA changes, but as post-transcriptional regulation is well known^2^,^3^,^31^, it is unsurprising to find that many are not. None of these analyses address the question of how consistently differentially expressed mRNAs translate into protein differences.

Here, we specifically examine mRNAs that are differentially expressed across experimental conditions and their relationships to their protein products. We calculate individual gene correlations across a time series in ovarian cancer xenograft models. We identify mRNAs that are differentially expressed between drug-treated and control samples. We compare those correlations where a gene’s mRNA was differentially expressed within that condition to those where the mRNA was not. We find that individual gene correlations of differentially expressed mRNA were significantly shifted to higher values, providing support for the assumption that differential mRNA expression has biological meaning.

## Results

**Figure 2:**
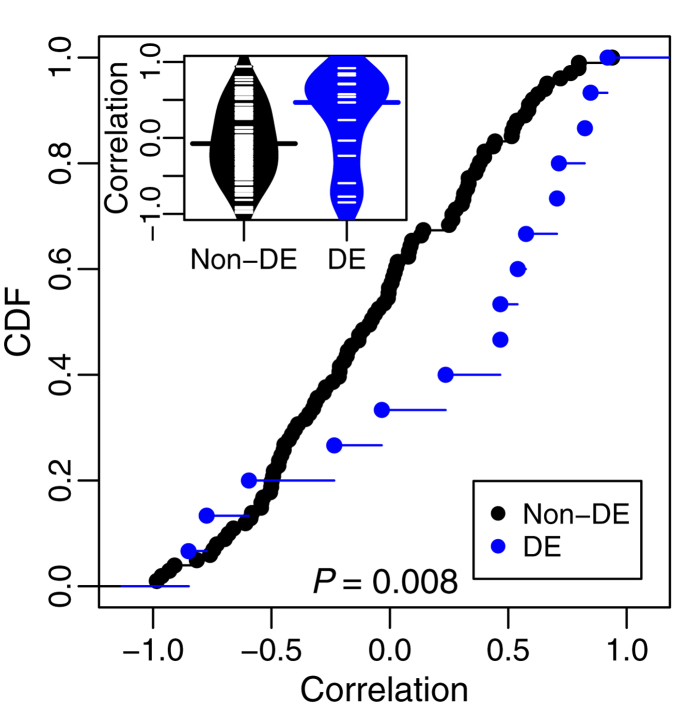
Distribution of mRNA-protein correlations for differentially expressed and non-differentially expressed mRNA profiles. The empirical cumulative distribution functions (CDF) over correlation coefficients between mRNA and protein values in a condition are plotted for differentially expressed mRNA profiles (DE) and non-differentially expressed mRNA profiles (Non-DE). Each individual correlation coefficient is based on n = 5 time points within a condition. Inset shows bean plots with ticks for each correlation value inside of density envelopes; bars represented medians for each group. Differentially expressed mRNA profiles are shifted significantly to higher correlations (Kolmogorov-Smirnov test, p = 0.008) and have a higher median (Wilcoxon test, p = 0.03).

We used mRNA expression data previously collected and published in Koussounadis *et al*.^32^ and newly collected protein data from the same experiment. Protein expression was measured by quantitative immunofluorescence as described in Methods and a representative image is illustrated in Supplementary Figure 1. Protein and mRNA expression measurements for twenty-nine genes were taken across a five-point time series (protein and mRNA ‘profiles’) in each of four different conditions, two xenograft models treated with two drug regimes (time-points ranging from 1 to 14 days after drug treatment, see Methods). Comparisons were made between the mRNA expression of each time point in each condition to that of pooled untreated controls. An mRNA was considered differentially expressed within a condition if any of the five time points showed a significant difference from control (at FDR < 0.05, see Methods; henceforth referred to as a differentially expressed mRNA profile). We calculated individual gene correlation coefficients between mRNA and protein profiles for each condition, using the five points in the time series. Compared to non-differentially expressed mRNA profiles, correlations for differentially expressed mRNAs profiles had a distribution significantly shifted towards higher values (Fig. 2; Kolmogorov-Smirnov test, p = 0.008) and a higher median (Fig. 2; Wilcoxon test, p = 0.03).

However, it is possible that this shift could be due to the fact that profiles containing differentially expressed mRNAs had a larger dynamic range than those without, leading to a higher signal-to-noise ratio and thus a tighter correlation. To evaluate this possibility, we implemented two models: (1) a simple model of random samples all having identical relationship between mRNA and protein, but with the mRNA drawn from either high- or low-variance distributions (equal to the variance of the differentially expressed and non-differentially expressed mRNA profiles, respectively) and calculating the same statistics as above, and (2) Monte Carlo shuffling of the mRNA-protein pairing for mRNA profiles to generate new profile correlation distributions against which to compare (see Methods for details of both).

Both models supported the significance of our results. The Kolmogorov-Smirnov and Wilcoxon statistics were significantly higher than those calculated from comparison between high- and low-variance samples from the simple model (Kolmogorov-Smirnov D, p = 0.008; Wilcoxon W, p = 0.022). The distribution of correlations for differentially expressed mRNA profiles was shifted to significantly higher values and had a higher median than those calculated from shuffled differentially expressed mRNA profiles (Kolmogorov-Smirnov test, p = 0.03; Wilcoxon test, p = 0.04). In contrast, the correlations for non-differentially expressed mRNA profiles were not significantly different from those calculated from the same profiles shuffled (Kolmogorov-Smirnov test, p = 0.29; Wilcoxon test, p = 0.25).

**Figure 3:**
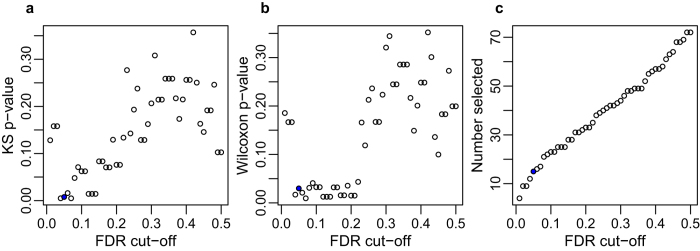
Relationship between FDR cut-off and difference in distributions of differentially expressed versus non-differentially expressed mRNA profiles. (**a**) The Kolmogorov-Smirnov p-value for comparison of distribution differences of differentially expressed versus non-differentially expressed mRNA profiles is plotted for each FDR cut-off used to define differentially expressed mRNA. (**b**) As in (**a**) for the Wilcoxon p-value for comparison of median differences. (**c**) Number of mRNA profiles selected as differentially expressed is plotted for each FDR cut-off. In all, the value for FDR < 0.05 as used in the main analysis is highlighted in blue.

Because of the relatively small size of our dataset, we further investigated the robustness of our results by repeating the analysis using different FDR cut-offs for selecting differentially expressed mRNA profiles, ranging from 0.01 to 0.50 (see Methods). If the phenomenon is robust and linked to the differential expression of mRNA, we would expect to see increasing levels of significance (lower p-values) as the FDR cut-off decreases. We found precisely this expected positive relationship between p-value and FDR cut-off for both the distribution and median comparisons (Fig. 3a,b; linear regressions: for Kolmogorov-Smirnov test, b = 0.41, t(48) = 6.10, p < 0.0001; for Wilcoxon test, b = 0.49, t(48) = 6.12, p < 0.0001). The exceptions to this rule were the three lowest FDR cut-off values, which had relatively high (and non-significant) p-values for both comparisons. This can be explained by a small sample size of less than ten differentially expressed mRNA profiles (Fig. 3c), leading to reduced power in the statistical test.

**Figure 4:**
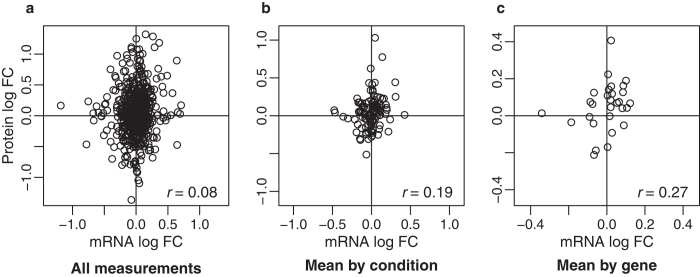
Genome-wide correlations for mRNA and protein expression. Scatterplots with associated correlation coefficients (r) for **(a)** all measurements taken (n = 579), **(b)** means by condition (n = 116), and **(c)** means by gene (n = 29).

For comparison with previous studies, we also explored genome-wide correlations on our data. From the genome-wide perspective, there was low correlation between all measured mRNA and protein expression levels (Fig. 4; r = 0.08, n = 579, p = 0.07). Consideration of mean values by condition (averaging across time points) or by gene (averaging across all measurements) resulted in higher correlations than using individual values, although only the correlation by condition was significant (Fig. 4; by condition r = 0.19, n = 116, p = 0.04, by gene r = 0.27, n = 29, p = 0.14). Genome-wide correlations considering only those measurements in differentially expressed mRNA profiles produced results similar to the above (Supplementary Fig. 2).

## Discussion

For the first time, we explore the relationship between an mRNA being differentially expressed and the mRNA-protein correlation for that gene. Compared to genes whose mRNA is not differentially expressed within a condition, we find that genes with differentially expressed mRNA have significantly higher correlations between mRNA and protein. This result provides support for the implicit assumption that differential mRNA expression reflects a difference between conditions at the functional level of proteins.

We found this significant correspondence despite also finding poor correlations from a genome-wide perspective, as have other studies. In fact, our correlations (r = 0.08 – 0.27) are on the low end^1^. It is interesting to note how the correlations increased as more averaging was performed. Most such studies collapse over several measurements to obtain one value per mRNA and per protein, either a mean^5^,^13^,^22^ or maximum^17^. Averaging across samples appears to approach capturing an overall correspondence of mRNA and protein levels across the genome, not reflecting specifics of dynamic responses to regulation. However, such dynamic changes are the question of interest when looking for differential expression.

Even against this background of seemingly low correspondence, our analysis revealed that the differentially expressed mRNA profiles were significantly shifted to higher correlations with their protein products than non-differentially expressed mRNA profiles. It was only a shift in distribution, not two distinct populations of profile correlations; however, this is to be expected. Within the non-differentially expressed mRNAs profiles, it would be expected that some have high correlations with their protein product for reasons unrelated to the experimental condition. Within the differentially expressed mRNAs profiles, there were some low and even negative correlations. Firstly it is always possible on a genome-scale analysis to have false positives; however, false positives could not account for all the low/negative profile correlations. It is also known that mRNA and protein profiles can be decoupled in time^15^,^30^, thus it is possible that correspondences for some of these correlations might have existed if different time scales were used. Finally, it may be the case that other levels of regulation overrode the transcriptional level, providing biological fine-tuning for the specific conditions encountered by the cells. It is notable that the non-differentially expressed profiles showed correlation distributions no different from those profiles randomised, whereas the differentially expressed profiles had significantly higher correlations than random. Thus, it appears differential expression does co-occur with tighter connection between mRNA and protein levels.

We show a subtle phenomenon: genes whose transcription is modified by experimental manipulation are more likely to show concordant protein expression across these same experimental conditions, compared to genes whose transcription is not strongly influenced by the experimental manipulation. This shift in likelihood supports the view that multiple levels of regulation act together to prepare a cell most effectively for the conditions it encounters. Transcription differences can be triggered by changes in the environment. These transcription changes can be modified or overridden by translational regulation. Rates of protein degradation, as well, will influence how closely a protein level tracks its transcript. Our results suggest that when the environment triggers a transcription difference, it is more likely that the same environment triggers concordant reactions for translational regulation for a gene—or perhaps *less* regulation at the translational level—compared to genes whose transcription is not being dynamically changed by these conditions. It may also be the case that protein degradation could be slowed or hastened to more quickly bring protein levels in line with changing transcript levels, compared to transcripts not currently in flux. The alternate possibility, that changes in mRNA expression are irrelevant to the protein composition of a cell—essentially meaningless—makes little biological sense. However, this concern has plagued the field since the low mRNA-protein correlations first began being reported. Thus, it is reassuring that our results show a real, if subtle, influence of differential mRNA expression on protein levels in our experimental system.

It may at first seem concerning that the profile correlation coefficients of all genes ranged the full –1 to 1 spectrum (r = −0.95 to 0.94, precisely). However, this wide range is perfectly in line with previous studies. Vogel *et al*.^30^ reported correlations across time points of a similar range to ours (r = −0.8 – 1). Chen *et al*.^22^ reported a narrower range (r = −0.47 – 0.44), but still centred around zero. One exception, Shankavaram *et al*.^28^, reported positive-shifted correlations (r = −0.1 – 0.8); however, these values were generated after several levels of filtering on the mRNA data for high autocorrelation and maximal correlation with protein, which could account for the positive shift compared to other studies. Many studies that examined individual mRNA-protein pairs across samples did not calculate correlation coefficients, but did report phenomena such as protein and mRNA changing in opposite directions^11^,^13^,^24^,^27^. Thus, as many previous studies have noted, protein and mRNA expression is often discordant.

It is encouraging that against this background, we were still able to discern a significant signal of increased concordance between mRNAs with differentially expressed profiles and their protein product, compared to mRNAs which were not actively regulated by the environment. Analysed from the same perspective as previous studies, our results are no different: overall low correspondence between mRNA and protein expression, implying strong contribution of post-transcriptional levels of regulation. However, by taking a different perspective—specifically addressing the implicit assumption of mRNA expression analysis that differential mRNA expression has some functional impact, most likely via protein expression—we found that differentially expressed mRNAs are more likely than non-differentially expressed mRNAs to translate into concordant behaviour at the protein level, giving confidence for the use of mRNA data for biological discovery.

Our study was relatively limited, with only 29 genes examined in 4 conditions for both protein and mRNA expression (116 correlations in total). However, our analysis using differing FDR cut-offs, which showed increasingly clear differences as cut-off decreases, indicates our result is not a statistical fluke of a particular set of correlations, but instead a general phenomenon within this experiment. Because there is no reason to suppose that our experimental system would produce greater mRNA-protein correspondence than any other system, we suggest this connection between dynamic regulation of mRNA and mRNA-protein correlation should be present in other systems. Thus, we believe that further and more extensive studies on this phenomenon are called for. Such studies should include multiple measurements of both mRNA and protein expression nested inside an experimental manipulation: this enables calculation of both individual-gene correlations (from the multiple measurements) and differential mRNA expression (from the experimental manipulation). Further exploration of the correlation between mRNA and protein under those conditions in which we apply differential mRNA expression analysis could shed more light on what information we can extrapolate from differentially expressed mRNAs.

## Methods

### Xenografts

Xenograft experiments are previously described in Koussounadis *et al*.^32^. Briefly, two ovarian cancer tumour models, OV1002 and HOX424^33^, were implanted subcutaneously in the flanks of adult female nu/nu mice and allowed to grow to 4-6 mm in diameter. The mice received one of two drug treatments via intraperitoneal injection on day 0, carboplatin (50 mg/kg) only or carboplatin (50 mg/kg) + paclitaxel (10 mg/kg), or were left untreated as controls. Xenografts were harvested from treated mice on days 1, 2, 4, 7, and 14, and from untreated controls on days 0, 1, 2, 7, and 14. The xenograft studies were conducted under a UK Home Office Project Licence in accordance with UK guidelines and regulations. Experiments were approved by the University of Edinburgh Animal Welfare and Ethical Review Body.

### mRNA expression

Full details of mRNA expression measurement and analysis are provided in Koussounadis *et al*^32^. Briefly, total mRNA was prepared from 10-50 mg of frozen tissue, and divided into two aliquots for two technical replicates per sample. Total RNA (0.5 mg) was amplified and biotinylated, diluted to 150 ng/ml, and hybridized to Illumina HT-12 BeadChips (Illumina, San Diego, CA, USA). This platform had been previously validated via PCR in a breast cancer xenograft study^34^. Expression values were processed with Bioconductor’s *lumi* package^35^. mRNA expression was measured in 3-4 biological replicates per time point per condition (except one which had 2 replicates; Supplementary Table 1); controls were only measured on days 0, 1, 7, and 14. Agreement among biological replicates was good as measured by Pearson correlation coefficients (mean 95% confidence interval r = 0.987 – 0.990; Supplementary Table 1).

### Protein expression

Protein was measured via immunofluorescence as previously described in Faratian *et al*^36^. Briefly, tissue microarrays were prepared from paraffin blocks of formalin-fixed xenograft material. Target proteins were chosen based on expectation of showing response in ovarian cancer treatment (e.g., representatives of MAPK, beta-catenin, ER, cell cycle, DNA-damage response pathways, among others). Antibodies for the proteins and conditions used are shown in Supplementary Table 2. Pan-cytokeratin antibody was used to identify tumour cells and normal epithelial cells, DAPI counterstain to identify nuclei, and Cy-5-tyramide detection for target for compartmentalised (tissue and subcellular) analysis of tissue sections. Monochromatic images of each TMA core were captured at 20X objective using an Olympus AX-51 epifluorescence microscope, and high-resolution digital images analysed by the AQUAnalysis software to generate AQUA (Automated quantitative analysis) expression scores for each sample (representative images shown in Supplementary Figure 1). Protein expression was measured in 3-8 biological replicates per time point per condition (except two, which had 1 and 2 replicates each; Supplementary Table 1). Agreement among biological replicates was good as measured by Pearson correlation coefficients (mean 95% confidence interval r = 0.938 – 0.958; Supplementary Table 1).

### Differential expression

Raw mRNA expression data were background corrected, variance stabilised transformed (VST) and robust spline normalised (RSN) using Bioconductor’s *lumi* package. AQUA protein expression scores were log-transformed with base 2. For both mRNA and protein expression, log fold-change values for each time point in each drug treatment condition were calculated by comparing mean expression levels across biological replicates to pooled controls for that tumour model using the Bioconductor package *limma*^37^. Both mRNA and protein expression exhibited similar dynamic ranges in log fold-change, from approximately –1 to 1. The output of *limma* was used to identify differentially expressed mRNAs, defined as those having FDR-adjusted p-values below 0.05. When evaluating varying FDR-cut offs, differentially expressed mRNAs were defined using FDR-adjusted p-values from 0.01 to 0.50 in steps of 0.01.

The mRNA dataset has been deposited to Gene Expression Omnibus (GEO) with accession number GSE49577. The protein dataset (raw AQUA scores and *limma*-produced log-fold change values) is provided in Supplementary Data 1.

### Correlations

Pearson correlation coefficients were calculated between mRNA and protein profiles for each drug treatment condition by correlating the log fold-change values for mRNA and protein expression across the five time points in each condition. For genes with multiple mRNA probes corresponding to a single protein, the probe with the highest average expression level across time points was used^35^. Genome-wide correlations were calculated on three different scales, by calculating Pearson correlation coefficients between mRNA and protein for: (1) all measurements taken, (2) mean across all time points in a condition, and (3) mean across all time points and conditions for each gene.

### Simple model of variance differences

To evaluate whether the higher correlation coefficients from differentially expressed mRNA profiles could be due to drawing from samples with higher variance, we implemented a simple model containing a single mRNA-protein correspondence, but drawing samples from high- and low-variance populations. Correspondence between mRNA and protein was modelled as
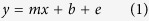


Where *y* is protein expression, *x* is mRNA expression, *m* is the slope, *b* is the intercept, and *e* is normally distributed noise. The values for *m* and *b* represent a single mRNA-protein correspondence identical for all pairs, and was calculated based on the corresponding values from a linear regression of log fold-change of mRNA against log fold-change of protein using all data. The noise *e* was generated by drawing from a normal distribution with mean equal to zero and standard deviation equal to the standard deviation of the residuals from the linear regression. The mRNA expression *x* was generated by drawing from a normal distribution with mean and standard deviation equal that of the mRNA log fold-change values for either: for high-variance, differentially expressed mRNA profiles; for low variance, non-differentially expressed mRNA profiles. Values for all variables used in the simple model are presented in Supplementary Table 3.

A single comparison was performed using this simple model as follows. For the high-variance correlations, one mRNA profile was created by randomly generating five samples from the high-variance distribution. The corresponding protein profile was calculated by determining protein expression for each of the group of five using equation (1). A correlation coefficient was then calculated between the mRNA and protein profiles. This process was repeated as many times as correlations existed for differentially expressed mRNA profiles. Similarly for the low-variance correlations, one mRNA profile was created by randomly generating five samples from the low-variance distribution. The corresponding protein profile was calculated by determining protein expression for each of the group of five using equation (1). A correlation coefficient was then calculated between the mRNA and protein profiles. This process was repeated as many times as correlations existed for non-differentially expressed mRNA profiles. Kolmogorov-Smirnov and Wilcoxon tests were then performed between the high-variance and low-variance correlation distributions.

The actual Kolmogorov-Smirnov and Wilcoxon statistics were compared to those generated from 100,000 repetitions of a single comparison from the simple model. For each test, the number of statistics greater than or equal to that of the actual statistic was counted to generate a p-value.

### Monte Carlo shuffling of profiles

Further tests for the significance of the higher correlation coefficients from differentially expressed mRNA profiles were calculated by shuffling the mRNA-protein pairing of profiles and calculating new correlations based on the shuffled profiles. A shuffling event shuffled the mRNA label for an entire profile, maintaining the order of time points within the profile: e.g., if mRNA X was swapped with mRNA Y, the first time point for mRNA X would be swapped with the first time point for mRNA Y, the second time point for mRNA X with the second time point for mRNA Y, and so on. This led to two different analyses.

In the first, mRNA-protein pairing of differentially expressed mRNA profiles were shuffled and new correlations calculated. Shuffling and correlation generation was repeated 1,000 times to provide a large distribution of shuffled profile correlations. The actual distribution of correlations for differentially expressed mRNA profiles was compared to this shuffled distribution using Kolmogorov-Smirnov and Wilcoxon tests.

The second was similar to the first, except that mRNA-protein pairing for non-differentially expressed mRNA profiles were shuffled. New correlations were calculated from these shuffled profiles. Shuffling and correlation generation were repeated 1,000 times to provide a large distribution of shuffled profile correlations. The actual distribution of correlations for non-differentially expressed mRNA profiles was compared to this shuffled distribution using a Kolmogorov-Smirnov and Wilcoxon tests.

## Additional Information

**How to cite this article**: Koussounadis, A. *et al*. Relationship between differentially expressed mRNA and mRNA-protein correlations in a xenograft model system. *Sci. Rep*. **5**, 10775; doi: 10.1038/srep10775 (2015).

## Supplementary Material

Supplementary Information

Supplementary Data 1

## Figures and Tables

**Figure 1:**
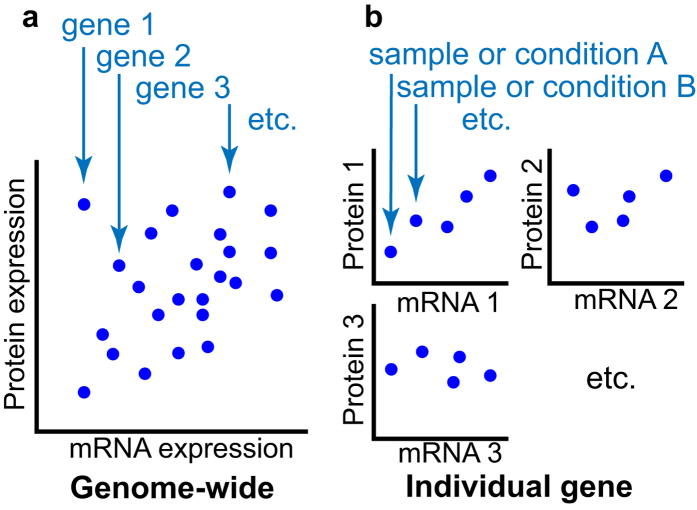
Genome-wide versus individual gene correlation analyses. **(a)** Most studies of mRNA-protein correspondence calculate a single correlation coefficient (or other correspondence metric) representing the correlation between mRNA expression and protein expression across all genes. Individual points upon which the correlation is calculated are a single value or an average of multiple samples/conditions, which do not necessarily have to be the same between mRNA and protein experiments. This correlation represents a general measure of how well mRNA and protein expression corresponds across the entire genome. **(b)** Studies of individual gene correspondence calculate a correlation coefficient (or other correspondence metric) for every gene, representing the correlation between the expression of a mRNA and its protein product across multiple samples or conditions. Individual points upon which the correlation is calculated are a single value or averaged samples for a single condition, which must correspond to the same sample or condition for both mRNA and protein measurements. This correlation represents how well the expression levels of a particular mRNA-protein pair correspond dynamically across the samples or conditions in the experiment. Three genes in (**a**) and (**b**) and two samples in (**b**) are highlighted for illustrative purposes only.
