# Supplementary material for: Relationship between differentially expressed mRNA and mRNA-protein correlations in a xenograft model system
Source: Sci Rep. 2015 Jun 8;5:10775. doi: 10.1038/srep10775 (PMC4459080; doi:10.1038/srep10775)
Supplement: Supplementary Information [file srep10775-s1.doc]

# Supplementary Information

**Relationship between differentially expressed mRNA and mRNA-protein correlations in a xenograft model system**

Antonis Koussounadis^1†^, Simon P. Langdon^2^, In Hwa Um^3^, David J. Harrison^3^, V. Anne Smith^1*^

^1^School of Biology, University of St Andrews, St Andrews, Fife, KY16 9TH, UK.

^2^Division of Pathology, University of Edinburgh, Edinburgh, EH4 2XU, UK.

^3^School of Medicine, University of St Andrews, St Andrews, Fife, KY16 9TF, UK.

^†^current address: Wolfson Wohl Cancer Research Centre, Institute of Cancer Sciences, University of Glasgow, Glasgow G61 1BD, UK.

^*^corresponding author: anne.smith@st-andrews.ac.uk

**
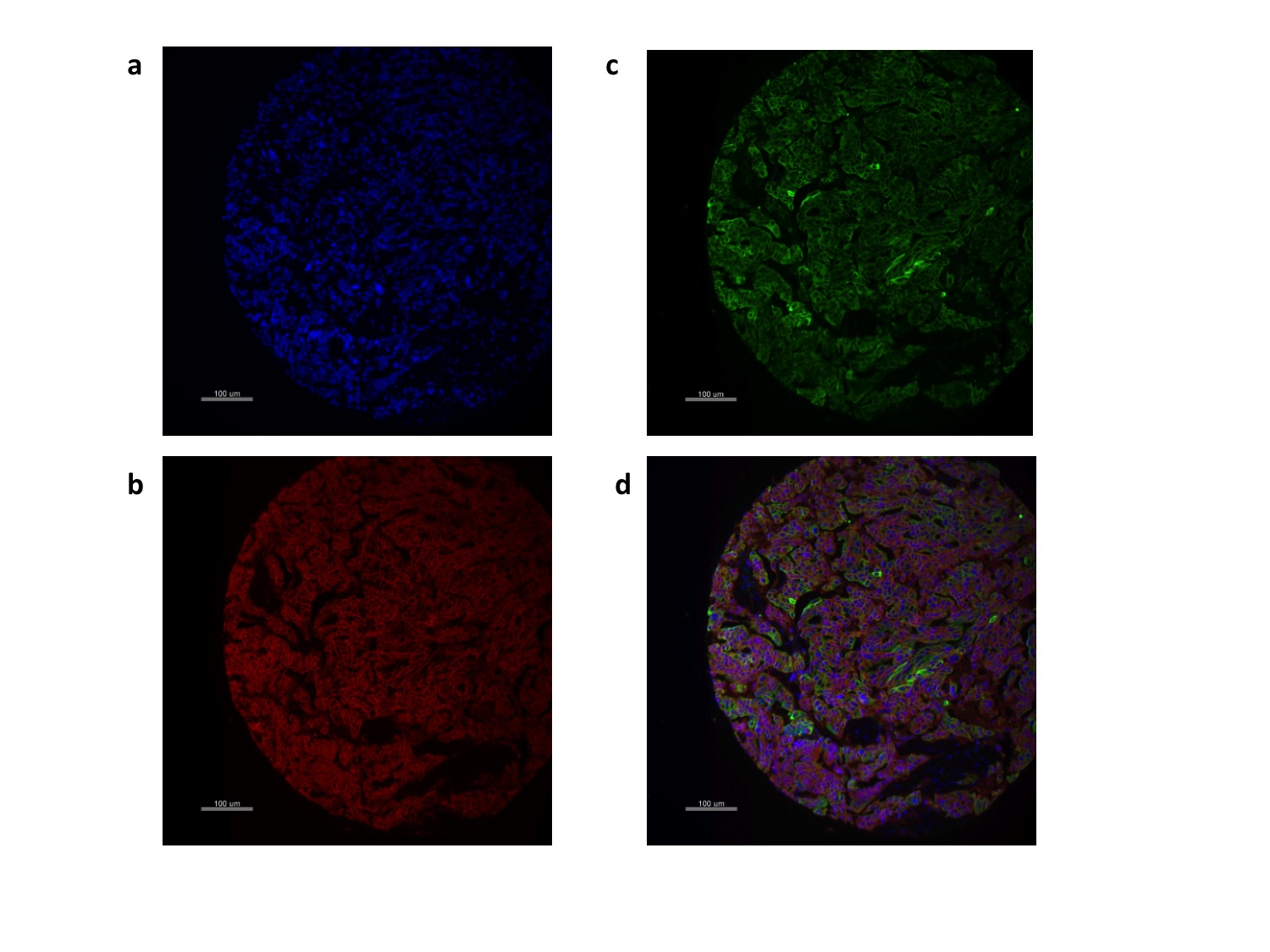
**

**Supplementary Figure 1. Representative immunofluorescence images of an OV1002 xenograft core demonstrating target protein expression.** In this example CDH1 (E-cadherin) expression is shown: **(a)** DAPI nuclear staining in blue, **(b)** cytokeratin staining in green, **(c)** CHD1 staining in red, and **(d)** composite image of all 3 markers. AQUA analysis software generates AQUA (Automated quantitative analysis) expression scores for each sample from the high-resolution digital image.

**Supplementary Figure 2. Genome-wide correlations for differentially expressed mRNA profiles superimposed on overall genome-wide correlations.** Scatterplots with associated correlation coefficients (r) for all genes (All), as in Fig. 1 of the main text are plotted as open circles, and only genes with differentially expressed mRNAs (DE) are plotted as blue-filled circles for **(a)** all measurements taken, **(b)** means by condition, and **(c)** means by gene. Genome-wide correlations for DE genes are higher than those calculated from all genes, although marginally significant or non-significant (all measurements, r = 0.23, n = 75, p = 0.05; by condition, r = 0.22, n = 15, p = 0.42; by gene, r = 0.32, n = 12, p = 0.30). A Monte Carlo analysis (see below) revealed that the increased correlation for genes with differentially expressed mRNA could be explained solely by the smaller sample size for the correlation (all measurements p = 0.079, by condition p = 0.446, by gene p = 0.409).

## Monte Carlo analysis of genome-wide correlations in Supplementary Figure 2

To evaluate whether the increased correlation coefficient for the genome-wide correlations of differentially expressed mRNAs could be explained by the smaller number of points in the correlation, a Monte Carlos analysis was performed. For the genome-wide correlation over means by condition and means by gene, a random selection from the appropriate set of all mean mRNA and protein expression values was taken of the same size as the number of differentially expressed genes (15 and 12, respectively), and a correlation coefficient calculated. This process was repeated 1,000 times, and the number of correlation coefficients equal to or greater than that for the differentially expressed genes was counted to generate a p-value.

For the genome-wide correlation over all measurements, to control for the fact that the correlation might be enhanced by selecting all measurements within in a condition, two analyses were performed. The first was done as for the means, selecting 75 random measurements from the set of all measurements; the second was done by first selecting 15 conditions, and then using all 75 measurements within these conditions. P-values were calculated as above, by repeating the process 1,000 times and counting the number of correlation coefficients equal to or greater than that for the differentially expressed selection. Results were similar for both analyses, and the p-value of the latter is presented above in the legend of Supplementary Figure 2.

**Supplementary Table 1. Number of biological replicates and repeatability for gene and protein expression.** Correlation coefficients were calculated for all possible pairs of biological replicates and averaged (Mean r) using expression values from all detected genes/proteins within each Tumour model/Treatment/Day.

| **Tumour model** | **Day** | **Treatment** | **mRNA expression** | | **Protein expression** | |
| --- | --- | --- | --- | --- | --- | --- |
|  |  |  | **Replicates** | **Mean *r*** | **Replicates** | **Mean *r*** |
| OV1002 | 0 | Control | 3 | 0.99 | 5 | 0.96 |
| OV1002 | 1 | Control | 3 | 0.99 | 5 | 0.91 |
| OV1002 | 2 | Control | - | - | 6 | 0.95 |
| OV1002 | 4 | Control | - | - | 6 | 0.95 |
| OV1002 | 7 | Control | 3 | 0.98 | 7 | 0.87 |
| OV1002 | 14 | Control | 3 | 0.99 | 8 | 0.94 |
| OV1002 | 1 | Carboplatin | 4 | 0.99 | 7 | 0.89 |
| OV1002 | 2 | Carboplatin | 4 | 0.99 | 7 | 0.96 |
| OV1002 | 4 | Carboplatin | 4 | 0.99 | 6 | 0.96 |
| OV1002 | 7 | Carboplatin | 4 | 0.99 | 7 | 0.95 |
| OV1002 | 14 | Carboplatin | 4 | 0.99 | 6 | 0.95 |
| OV1002 | 1 | Carboplatin-Paclitaxel | 4 | 0.99 | 7 | 0.91 |
| OV1002 | 2 | Carboplatin-Paclitaxel | 4 | 0.99 | 6 | 0.97 |
| OV1002 | 4 | Carboplatin-Paclitaxel | 4 | 0.99 | 5 | 0.88 |
| OV1002 | 7 | Carboplatin-Paclitaxel | 4 | 0.99 | 7 | 0.92 |
| OV1002 | 14 | Carboplatin-Paclitaxel | 4 | 0.99 | 4 | 0.94 |
| HOX424 | 0 | Control | 3 | 0.98 | 8 | 0.97 |
| HOX424 | 1 | Control | 3 | 0.99 | 6 | 0.96 |
| HOX424 | 2 | Control | - | - | 5 | 0.98 |
| HOX424 | 4 | Control | - | - | 5 | 0.98 |
| HOX424 | 7 | Control | 3 | 0.99 | 5 | 0.96 |
| HOX424 | 14 | Control | 3 | 0.99 | 5 | 0.96 |
| HOX424 | 1 | Carboplatin | 4 | 0.99 | 3 | 0.98 |
| HOX424 | 2 | Carboplatin | 4 | 0.98 | 2 | 0.98 |
| HOX424 | 4 | Carboplatin | 3 | 0.99 | 3 | 0.98 |
| HOX424 | 7 | Carboplatin | 4 | 0.99 | 5 | 0.96 |
| HOX424 | 14 | Carboplatin | 4 | 0.98 | 5 | 0.97 |
| HOX424 | 1 | Carboplatin-Paclitaxel | 4 | 0.99 | 3 | 0.94 |
| HOX424 | 2 | Carboplatin-Paclitaxel | 2 | 0.98 | 1 | - |
| HOX424 | 4 | Carboplatin-Paclitaxel | 4 | 0.99 | 4 | 0.96 |
| HOX424 | 7 | Carboplatin-Paclitaxel | 4 | 0.99 | 3 | 0.98 |
| HOX424 | 14 | Carboplatin-Paclitaxel | 4 | 0.99 | 4 | 0.96 |
|  |  |  | **total:**  101 | **95% CI:** 0.987-0.990 | **total:**  166 | **95% CI:**  0.938-0.958 |

**Supplementary Table 2. Proteins and antibodies used.** Antibody supplier and dilution used for each protein (gene correspondence also shown).

| **Gene name** | **Entrez Gene id** | **Protein name** | **Source** | **Catalog No.** | **Dilution** |
| --- | --- | --- | --- | --- | --- |
| AKT1 | 207 | AKT | Cell Signaling | Ab4685 | 1 in 200 |
| BRCA1 | 672 | BRCA1 | Eurogentec | 75460 | 1 in 70 |
| CCNB1 | 891 | CyclinB1 | Epitomics | 1495-1 | 1 in 50 |
| CCND1 | 595 | CyclinD1 | Dako | M3635 | 1 in 100 |
| CDH1 | 999 | Ecadherin | BD sciences | 610181 | 1 in 1500 |
| CDK2 | 1017 | CDK2 | Cell Signaling | 2546 | 1 in 100 |
| CDKN1B | 1027 | P27 | Cell Signaling | 2552 | 1 in 100 |
| CLDN7 | 1366 | Claudin7 | Abcam | AB27487 | 1 in 200 |
| CTNNB1 | 1499 | Bcatenin | BD sciences | 610153 | 1 in 500 |
| EGFR | 1956 | EGFR | Invitrogen | 28-0005 | 1 in 50 |
| ERBB2 | 2064 | HER2 | Dako | A0485 | 1 in 400 |
| ERBB3 | 2065 | HER3 | Dako | M7297 | 1 in 50 |
| ERCC1 | 2067 | ERCC1 | Labvision | MS-671-P0 | 1 in 600 |
| ESR1 | 2099 | ER | Dako | 7047 | 1 in 50 |
| HIF1A | 3091 | HIF1alpha | Cell Signaling | 3176 | 1 in 25 |
| IGFBP3 | 3486 | IGFBP3 | Abcam | Ab4248 | 1 in 100 |
| MAPK1 | 5594 | ERK | Cell Signaling | 9107 | 1 in 250 |
| MET | 4233 | MET | Eurogentec | 75551 | 1 in 400 |
| MLH1 | 4292 | MLH1 | Leica | NCL-L-MLH1 | 1 in 100 |
| MSH2 | 4436 | MSH2 | Invitrogen | 33-7900 | 1 in 50 |
| MSH6 | 2956 | MSH6 | Leica | NCL-L-MSH6 | 1 in 250 |
| MYC | 4609 | Myc | Eurogentec | 75355 | 1 in 70 |
| NCOA3 | 8202 | AIB1 | BD sciences | 61105 | 1 in 50 |
| PGR | 5241 | PgR | Dako | 3569 | 1 in 50 |
| PMS2 | 5395 | PMS2 | Leica | NCL-L-PMS2 | 1 in 250 |
| PTEN | 5728 | PTEN | Cell Signaling | 9559 | 1 in 200 |
| RPS6 | 6194 | S6 | Cell Signaling | 2217 | 1 in 100 |
| SERPINE1 | 5054 | PAI | BD Sciences | P612024 | 1 in 200 |
| VIM | 7431 | Vimentin | Sigma | V6630 | 1 in 400 |

**Supplementary Table 3. Variable values for simple model.** The values for variables in generating samples from *y = mx + b + e*, where *e* ~ *N*(*µ_e_*, *σ_e_*), and *x* ~ *N*(*µ_d_*, *σ_d_*) for high variance samples and *x* ~ *N*(*µ_n_*, *σ_n_*) for low variance samples.

| Variable | Symbol | Value |
| --- | --- | --- |
| Slope | m | 0.05374 |
| Intercept | b | 0.14531 |
| Mean of error | µ_e_ | 0 |
| Standard deviation of error | σ_e_ | 0.3731844 |
| Mean of high variance mRNA | µ_d_ | 0.05004508 |
| Standard deviation of high variance mRNA | σ_d_ | 0.3557132 |
| Mean of low variance mRNA | µ_n_ | -0.01059483 |
| Standard deviation of low variance mRNA | σ_n_ | 0.1559115 |

**Supplementary Data 1. Protein data.** Raw AQUA scores and log-fold change values produced by Bioconductor’s package *limma* are provided in the accompanying Excel spreadsheet.
